# Successful fluoroless radiofrequency catheter ablation of supraventricular tachycardia during pregnancy

**DOI:** 10.1002/ccr3.1623

**Published:** 2018-05-29

**Authors:** Georgy Kaspar, Kumar Sanam, Sujana Gundlapalli, Dipak Shah

**Affiliations:** ^1^ Department of Cardiology Providence‐Providence Park Hospital/Michigan State University College of Human Medicine Southfield MI USA; ^2^ Department of Cardiac Electrophysiology Providence‐Providence Park Hospital/Michigan State University College of Human Medicine Southfield MI USA

**Keywords:** ablation, radiation, supraventricular tachycardia

## Abstract

Even in the absence of underlying heart disease, pregnancy is known to increase susceptibility supraventricular tachycardia (SVT). This brings a management challenge, mainly due to concerns about pharmacotherapy and radiation to the fetus. This case highlights the capability of using fluoroless mapping technologies to treat refractory arrhythmia cases safely and successful.

## 1 INTRODUCTION

**Figure 1:**
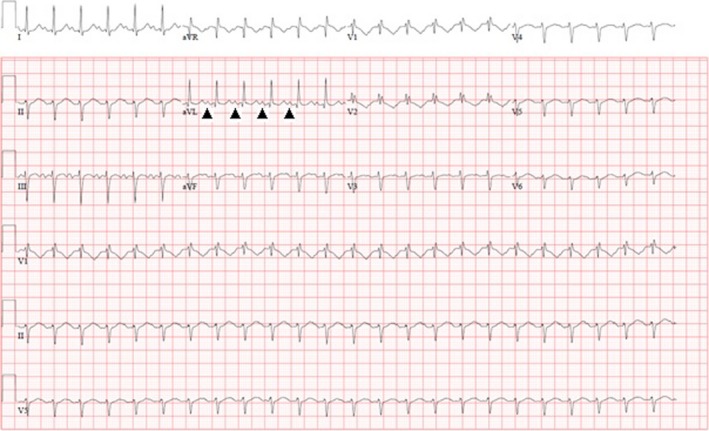
EKG shows supraventricular tachycardia with long RP interval. Arrow heads point to the P waves

Even in the absence of underlying heart disease, pregnancy is known to increase susceptibility to a variety of arrhythmias. Recurrent supraventricular tachycardia (SVT) during pregnancy may present a difficult management problem, mainly due to concerns about pharmacotherapy and radiation to the fetus. We report a case of 34‐year‐old woman in her 11th week of pregnancy with recurrent symptomatic refractory SVT who underwent a successful fluoroless ablation. Her electrocardiogram (Figure 1) demonstrated a long RP SVT with a heart rate of 180 beats per minute. A cardioversion with anesthesia was transiently successful; however, she had shortly a recurrence of her tachycardia. A repeat cardioversion with an Amiodarone bolus was also unsuccessful. With Sotalol the SVT was no longer incessant, but the patient continued to show frequent long periods of SVT or salvos of premature atrial contractions (PACs). Given her continued instability, she was referred for an electrophysiology study and ablation. To minimize the radiation risk to the fetus we attempted a fluoroless ablation and we were able to terminate her PACs. This case highlights the capability of using only mapping technologies to treat challenging arrhythmia cases safely and successful.

Even in the absence of underlying heart disease, pregnancy is known to increase susceptibility to a variety of arrhythmias.^1^ Recurrent supraventricular tachycardia (SVT) during pregnancy may present a difficult management problem, mainly due to concerns about pharmacotherapy and radiation to the fetus.^2^

## 2 CASE

We report a case of 34‐year‐old woman in her 11th week of pregnancy with recurrent symptomatic refractory SVT who underwent a successful fluoroless ablation.

Her electrocardiogram (Figure 1) demonstrated a long RP SVT with a heart rate of 180 beats per minute. She failed vagal maneuvers and adenosine administration and was started on a Diltiazem and Esmolol drip. Although her rate decreased to 120 beats per minute she became hypotensive. A cardioversion with anesthesia was transiently successful; however, she had shortly a recurrence of her tachycardia. A repeat cardioversion with an amiodarone bolus was also unsuccessful. With sotalol the SVT was no longer incessant, but the patient continued to show frequent long periods of SVT or salvos of premature atrial contractions (PACs). Given her continued instability, she was referred for an electrophysiology study and ablation.

**Figure 2:**
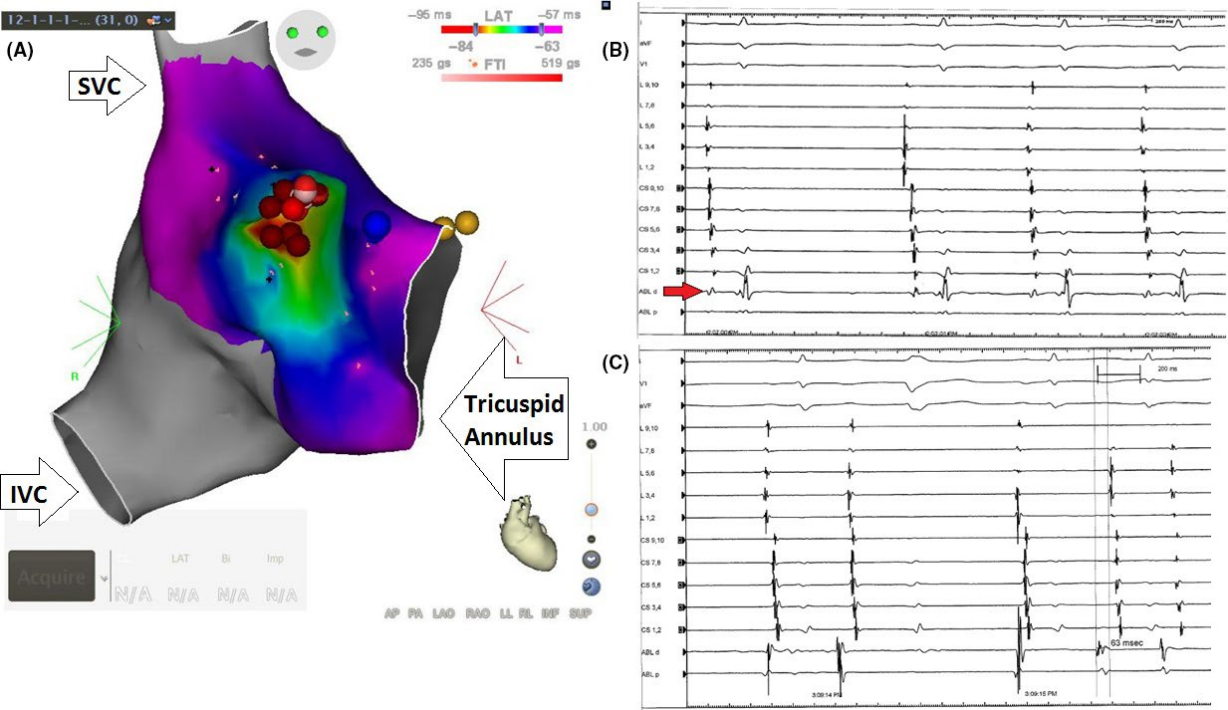
A, CARTO mapping of the right atrium. The red dots represent the area of ablation where the origin of the PACs is identified using the mapping technique (red background). SVC‐ superior vena cava. IVC‐ inferior vena cava. B, An intracardiac electrogram shows frequent PACs from crista terminalis before ablation. Looking to ABL d electrogram (arrow) from the ablation catheter, you can identify the first sinus beat, that is, followed by 3 ectopic beats with shorter PR interval. C, An intracardiac electrogram shows successful ablation of PACs

**Figure 3:**
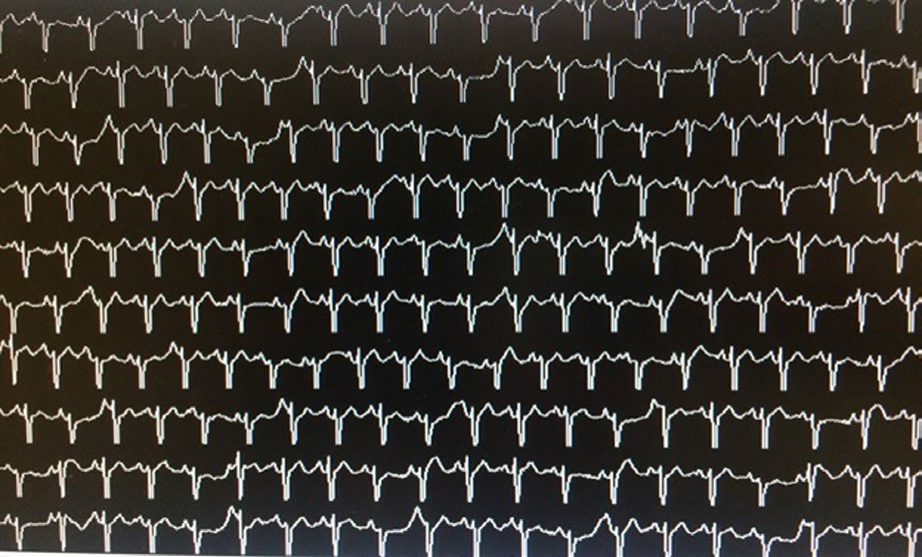
Telemetry after ablation showing absence of PACs

To minimize the radiation risk to the fetus we attempted a fluoroless ablation. Three dimensional (3D) electro‐anatomical mapping (CARTO 3 Version 4 software, Biosense Webster, Irwindale, California) was created with an irrigated contact force sensing catheter (Figure 2A). With the right atrial (RA) geometry including the coronary sinus delineated, a steerable decapolar catheter was placed in the coronary sinus (CS) using our map as a reference. Although with sedation the patient was not in tachycardia, she was having frequent salvos of PACs (Figure 2B) with proximal to distal activation on the CS tracings. The differential for the origin of the tachycardia was thought to be from the right‐sided pulmonary veins, superior vena cava (SVC) tachycardia or crista terminalis (CT). The patient therefore had another decapolar catheter placed along the CT with its most distal pole in the SVC. Isoproterenol of 2 mcg/min was administered; however, we could still not induce the tachycardia. Activation mapping of these PACs consistently demonstrated earliest activity on the anterior‐superior portion of the CT. At this location, we had poor contact force and therefore the short sheath was exchanged for a Schwartz Right 0 (SR0) sheath. Prior to ablation high output pacing was performed and demonstrated no phrenic nerve capture. With the sheath, we could obtain a contact force between 10‐20 g and ablation at 30‐35 Watts resulted in immediate termination of the PACs (Figure 2C). The Holter at the end of the case demonstrated no further PACs (Figure 3).

## 3 DISCUSSION

An exacerbation of arrhythmias during pregnancy is common secondary to an increase in atrial or ventricular premature beats. Symptoms are usually aggravated with the associated hormonal and hemodynamic changes. This carries a higher risk of adverse fetal complications, independent of other maternal and fetal risk factors.^3^

Supraventricular tachycardia is usually divided into 2 categories by assessing the relationship of the P waves to the QRS complex (R waves). Our patient had a long RP interval SVT. The differential diagnosis of this first category contains the following: atrial tachycardia, sinus tachycardia, sinus node reentry, atrial flutter, atrioventricular reentrant tachycardia, atypical (fast‐slow) atrioventricular nodal reentrant tachycardia and Permanent junctional reciprocating tachycardia due to a slowly conducting retrograde accessory pathway. The second category is short RP interval SVT with following differential diagnosis: typical atrioventricular nodal reentrant tachycardia, atrioventricular reentrant tachycardia using accessory pathways, atrial tachycardia with long first‐degree AV block and atrial tachycardia originating from the os of the coronary sinus and junctional tachycardia.

Non‐pharmacological approach with vagal maneuvers is considered the first benign attempt in terminating SVT. Pharmacological approach with Adenosine is usually effective, whereas, monitoring the fetal heart rate is recommended. When Adenosine fails, other antiarrhythmic can be indicated like Beta blockers and calcium channel blockers. Amiodarone is considered contraindicated because of its potential teratogenic effects. Whereas, digoxin has no reported fetal toxicities during any gestational stage. Electrical cardioversion is benign because it only delivers a minimal amount of current to the fetus.^4^

Recent ACC/AHA/ESC guidelines for management of SVT list catheter ablation as a reasonable approach in pregnant patients with highly symptomatic, recurrent, drug‐refractory SVT with efforts toward minimizing radiation exposure. This is considered a IIb indication with a level of evidence C (primarily based on expert consensus).^5^ Szumowski et al^6^ showed that in the setting of malignant, drug‐resistant arrhythmias, therapeutic ablation may be considered with no or minimal radiation exposure during pregnancy.

Exposure to less than 5 rad [50 mGy] has not been associated with an increase in fetal anomalies or pregnancy loss.^7^ However, the risk of radiation exposure to the fetus is a real concern with catheter ablation given that high‐dose ionizing radiation has been linked to excess malignancy and congenital malformations.^8^

In general, ECG monitoring after catheter ablation is essential. Patients may sooner or later develop other types of arrhythmia. Antiarrhythmic and antithrombotic drug prescription can be considered in cases with high‐risk factors but usually are not recommended in young patients with no risk factors. There are insufficient data on long‐term outcome with respect to the occurrence of atrial arrhythmias in patients after successful ablation.

## 4 CONCLUSION

This case highlights the capability of using only mapping technologies to treat challenging arrhythmia cases safely and successfully.

## CONFLICT OF INTEREST

None declared.

## AUTHORSHIP

GK: participated in the design and drafting the manuscript. KS: participated in the design and drafting the manuscript. SG: participated in the design and drafting the manuscript. DS: revised the manuscript.

## References

[1] Shotan A , Ostrzega E , Mehra A , et al. Incidence of arrhythmias in normal pregnancy and relation to palpitations, dizziness, and syncope. Am J Cardiol. 1997;79:1061‐1064. 9114764 10.1016/s0002-9149(97)00047-7

[2] Kanjwal Y , Kosinski D , Kanj M , Thomas W , Grubb B . Successful radiofrequency catheter ablation of left lateral accessory pathway using transseptal approach during pregnancy. J Interv Card Electrophysiol. 2005;13:239‐242. 16177852 10.1007/s10840-005-2493-1

[3] Silversides CK , Harris L , Haberer K , et al. Recurrence rates of arrhythmias during pregnancy in women with previous tachyarrhythmia and impact on fetal and neonatal outcomes. Am J Cardiol. 2006;97:1206‐1212. 16616027 10.1016/j.amjcard.2005.11.041

[4] Rotmensch HH , Elkayam U , Frishman W . Antiarrhythmic drug therapy during pregnancy. Ann Intern Med. 1983;98:487‐497. 6132574 10.7326/0003-4819-98-4-487

[5] Page R , Joglar J , Caldwell MA , et al. 2015 ACC/AHA/HRS Guideline for the management of adult patients with supraventricular tachycardia. JACC ISSN 0735‐1097

[6] Szumowski L , Szufladowicz E , Orczykowski M , et al. Ablation of severe drug‐resistant tachyarrhythmia during pregnancy. J Cardiovasc Electrophysiol. 2010;21:877‐882. 20158563 10.1111/j.1540-8167.2010.01727.x

[7] Damilakis J , Theocharopoulos N , Perisinakis K , et al. Conceptus radiation dose and risk from cardiac catheter ablation procedures. Circulation. 2001;104:893‐897. (did not use it, it says that only 1 mG is the average radiation we use in fluoro radiation!!) 11514375 10.1161/hc5790.094909

[8] McCollough CH , Schueler BA , Atwell TD , et al. Radiation exposure and pregnancy: when should we be concerned? Radiographics. 2007;27:909‐917. 17620458 10.1148/rg.274065149

